# Public holidays increased the transmission of COVID-19 in Japan, 2020-2021: a mathematical modelling study

**DOI:** 10.4178/epih.e2024025

**Published:** 2024-01-22

**Authors:** Jiaying Qiao, Hiroshi Nishiura

**Affiliations:** School of Public Health, Kyoto University, Kyoto, Japan

**Keywords:** Vacation, SARS-CoV2, Epidemiology, Effective reproduction number, Causal inference

## Abstract

**OBJECTIVES:**

Although the role of specific holidays in modifying transmission dynamics of infectious diseases has received some research attention, the epidemiological impact of public holidays on the transmission of coronavirus disease 2019 (COVID-19) remains unclear.

**METHODS:**

To assess the extent of increased transmission frequency during public holidays, we collected COVID-19 incidence and mobility data in Hokkaido, Tokyo, Aichi, and Osaka from February 15, 2020 to September 30, 2021. Models linking the estimated effective reproduction number (*R_t_*) with raw or adjusted mobility, public holidays, and the state of emergency declaration were developed. The best-fit model included public holidays as an essential input variable, and was used to calculate counterfactuals of *R_t_* in the absence of holidays.

**RESULTS:**

During public holidays, on average, *R_t_* increased by 5.71%, 3.19%, 4.84%, and 24.82% in Hokkaido, Tokyo, Aichi, and Osaka, respectively, resulting in a total increase of 580 (95% confidence interval [CI], 213 to 954), 2,209 (95% CI, 1,230 to 3,201), 1,086 (95% CI, 478 to 1,686), and 5,211 (95% CI, 4,554 to 5,867) cases that were attributable to the impact of public holidays.

**CONCLUSIONS:**

Public holidays intensified the transmission of COVID-19, highlighting the importance of considering public holidays in designing appropriate public health and social measures in the future.

## GRAPHICAL ABSTRACT

[Fig f4-epih-46-e2024025]

## Key Message

• The number of COVID-19 cases increased during public holidays in Japan.

• The increase may have occurred because of elevated mobility rate and altered contact behaviours.

• The effect of holidays varied by prefecture in Japan.

## INTRODUCTION

As of May 8, 2023, the cumulative number of confirmed coronavirus disease 2019 (COVID-19) cases in Japan was 33,803,572. At this time, COVID-19 had been legally downgraded to a Category V Infectious Disease, alongside seasonal influenza and other common respiratory viral infections [1,2]. The change of category corresponded to the discontinuation of a series of public health and social measures, including governmental requests for movement restrictions and maintaining social distance [2]. After 3 years without mass gathering events, some parades and ceremonies held during public holidays restarted [3]. Although the governmental response to COVID-19 has clearly shifted to a post-vaccination phase, public concerns over COVID-19 transmission, limited healthcare capacity, and complications and mortality arising from the pandemic have not completely waned. Despite the reopening of society, it remains important to consider science-based countermeasures.

Previous studies have reported that public holidays are an important driver of the transmission of infectious diseases, as exemplified by the transmission of Middle East respiratory syndrome during Hajj and the COVID-19 pandemic [4]. First, the frequency of high-risk behaviours, such as indoor gatherings and inconsistent mask-wearing, has been found to increase over holidays [5,6]. An observational study in Bangkok, Thailand found that people tended to reduce mask-wearing during holidays [7]. Second, human mobility patterns typically change during holidays, such as an increased tendency to travel longer distances than usual [8]. Long-distance travel between different communities can accelerate the spatial spread of COVID-19 [9,10]. A published study in Spain showed that movement to tourism spots increased significantly during holidays [11]. Meanwhile, a study conducted in Italy reported that the daily number of new COVID-19 infections abruptly increased after summer holidays in holiday destinations, and a nationwide upsurge in infection numbers was observed after a special Italian holiday in August 2020 [12].

Despite the significance of public holidays in constructing countermeasures to combat disease transmission, few published studies have explored the effects of public holidays, except as a covariate for adjustment in regression models designed to explore the role of other variables in modifying transmission [13,14]. Moreover, while a few published studies have demonstrated the importance of holidays in increasing transmission in Japan, those studies have focused on specific single holidays such as New Year (NY)’s Day or Coming-of-Age Day using ground-based approaches from field epidemiology [15,16]. A comprehensive statistical analysis of other public holidays and a causal inference of their role in increasing the transmission of COVID-19 is warranted. Such an analysis should also consider technical pitfalls in analysing the transmission dynamics as a function of time of infection, rather than the time of illness onset or time of reporting, to avoid bias caused by time delay [17,18].

The objective of the current study was to estimate the degree to which the transmission of COVID-19 increased during public holidays in Japan and infer the causal impact of holidays in increasing the incidence of the disease. We constructed a mathematical model to link public holidays and the effective reproduction number (*R_t_*) in Japan from 2020 to 2021.

## MATERIALS AND METHODS

### Epidemiological data

Because the number of cases in Japan remained low during the first 2 years of the pandemic in 2020-2021, we chose 4 urban prefectures (Hokkaido, Tokyo, Aichi, and Osaka) for the analysis. Tokyo is the capital of Japan, and has the highest population density. Hokkaido, Aichi, and Osaka are three urban prefectures in eastern, middle, and western Japan. We analysed the daily number of confirmed COVID-19 cases in these prefectures using a dataset from the Ministry of Health, Labour and Welfare in Japan [1]. The data were aggregated by the date of report and prefecture. To cover a sufficient number of public holidays, the study period was set from February 15, 2020 to September 30, 2021. The timing of the end of our study period approximately corresponded to the end of the fifth wave of the COVID-19 pandemic in Japan, and the time at which primary series vaccination among older people was in the process of completion. After the study period, the incidence was greatly reduced by vaccination, and, subsequently, the Omicron variant started to circulate in late 2021 [19]. During the study period, there were 60,053, 365,563, 108,499, and 198,223 confirmed COVID-19 cases in Hokkaido, Tokyo, Aichi, and Osaka, respectively. We back-projected the date of infection from the date of report, then computed the *R_t_* using the renewal process model [20,21].

### Explanatory variables

Previous studies have reported a strong correlation between mobility and transmissibility, and public health and social measures aiming to reduce *R_t_* have mainly targeted human mobility [22-25]. Thus, mobility was selected as an explanatory variable of *R_t_*, and to adjust for stringent control, the state of emergency declaration was also used as an explanatory variable. Because analyses of the effect of temperature on transmission rates have yielded conflicting results in previous studies, particularly in describing long-term dynamics, we decided to avoid its use [22]. In addition to these variables, we prepared consecutive public holidays as a variable of interest.

#### Holidays

Japan has 16 national public holidays, which were established by the Public Holiday Law [3]. These holidays are typically 1 day long, but when they occur on Monday or Friday, they are added to the weekend and considered as 3-day or 4-day consecutive holidays. When a holiday takes place during weekends, there is typically a substitute holiday on a weekday (this substitute holiday is usually taken on the subsequent Monday). There are 3 special public holidays and vacations: (1) NY’s Day is an official 1-day holiday, but holidays usually last for 5 days from the end of the year (from 30 December) to the start of the next year (to 3 January), (2) Golden Week (GW) is composed of 3 single holidays, meaning that a weekend will commonly last for more than 5 days, and (3) *Obon* is not an official public holiday, but is a traditional vacation time in Japan, during which people typically take days off work to spend time in their home town while a series of parades are held. In our study, we assumed that observable changes in movement only happened during consecutive holidays. We used a binary variable (0 or 1) to represent the presence of a consecutive holiday on a calendar time scale. The holiday data used in the present study are shared as Supplementary Material 1.

#### Mobility

The mobility data were assessed using Google COVID-19 Community Mobility Reports, which charted movement trends over time by geography, across 6 different categories of places [26]. The category of “retail & recreation” reflects mobility in places like restaurants and shopping centres, which are considered to be highrisk settings for COVID-19 transmission [27]. The daily population size at a specified place was compared with that on the baseline day and reported as the relative change. The baseline day represents a normal value for that day of the week, corresponding to the median value from the 5 weeks from January 3, 2020 to February 6, 2020. According to the guidelines from Google, the first step for examining a change caused by community responses to COVID-19 is to mark off public holidays and vacations. In the current study, we used raw mobility data, and, moreover, mobility was adjusted by removing the original mobility data during public holidays and imputing the alternative mobility data using linear interpolation. The interpolation procedure assumed a linear relationship between the known data points (i.e., mobility before and after the public holiday) and estimated corresponding values within the given range. The imputation was performed using the “imputeTS” package in R.

#### The state of emergency

According to the special measures law of COVID-19 control in Japan, the government declared a state of emergency when the number of infected cases increased rapidly and the caseload demand in hospitals became excessive [28]. The exact period of the state of emergency differed between prefectures. During the period of the state of emergency, a series of public health and social measures were implemented, including shortening the opening hours of restaurants, suspending mass gathering events, and a request for people to exercise self-restraint and stay home. We used a binary variable (0 or 1) to represent the period during which the state of emergency was declared.

### Statistical analysis

First, we reconstructed the mobility dataset of each prefecture by deleting data during holiday periods, then used linear interpolation to impute those gaps. The reconstructed mobility dataset is referred to as “adjusted mobility” throughout this article. Second, we attempted different combinations of variables to model the relationship between *R_t_* and holidays. To ensure reliability, we examined the proposed model for all 4 prefectures. Third, we chose the best model to predict *R_t_*, and then produced a counterfactual scenario. We calculated the incidence of the counterfactual scenario and subtracted it from the observed incidence. All data analyses were conducted using R version 4.2.2 (R Foundation for Statistical Computing, Vienna, Austria).

#### Model descriptions

To link public holidays with *R_t_*, we used a log-linear type model as below:


(1) $$R\left(t\right)=R*\exp\left(a*h\left(t\right)+b*m\left(t\right)+c*e\left(t\right)\right)$$

In this formula, *R(t)* is the effective reproduction number at a calendar date *t, R* is the reproduction number at baseline and acts as a scaling factor constant, *h(t)* represents a dichotomous variable indicating consecutive public holidays, *m(t)* represents the percentage of change in Google mobility on day *t*, and *e(t)* represents a dichotomous variable adjusting for whether the government declared the state of emergency on day *t*. The parameters *a, b*, and *c* are estimated using the maximum likelihood method. We tested 5 possible models: model 1 (state of emergency+raw mobility), model 2 (state of emergency+adjusted mobility), model 3 (state of emergency+holiday), model 4 (state of emergency+raw mobility+ holiday), and model 5 (state of emergency+adjusted mobility+ holiday). In model 1, we used the state of emergency and raw mobility to explain changes in *R_t_*, expecting a positive association between mobility and transmission, and a negative association between the state of emergency and transmission. In model 2, we replaced the raw mobility data with adjusted mobility data that eliminated holiday-caused peaks from mobility. In model 3, we only included state of emergency and holiday to explain changes in *R_t_*. We then considered using models 4 and 5, which included the state of emergency, public holidays, and either raw mobility or adjusted mobility. These 5 models were designed to capture different causal pathways of the effects of holidays on influencing transmission dynamics: model 1 focused on the indirect effect on transmission as mediated by mobility, model 2 explored the possibility that holidays would have no effect on transmission at all, model 3 focused on the direct effect of holidays on transmission without adjusting for mobility, model 4 explored the direct and indirect effects of holidays on transmission, with the indirect effect mediated by mobility, and model 5 concentrated on the direct effect of holidays on transmission, adjusting for general trends in mobility. Maximum likelihood estimation was performed to estimate parameters, and the 95% confidence intervals (CIs) for each parameter were derived from the parametric bootstrap method (i.e., a Laplace approximation normal distribution from 10,000 samples). We assumed that the distribution of confirmed cases was sufficiently captured by a Poisson distribution, and the likelihood function was:


(2) $$L\left(i\left(t\right)\right)=\prod_{t=1}^{T}\frac{E{\left(i\left(t\right)\right)}^{t\left(t\right)}exp\left(-E\left(i\left(t\right)\right)\right)}{i\left(t\right)!}$$

In formula (2), *i(t)* is the observed daily number of cases on day *t*, *T* is the total number of observation days, *E(i(t))* is the expected value of the number of cases that is parameterised by:


(3) $$E\left(i\left(t\right)\right)=R\left(t\right)\sum_{\tau -1}^{t-1}i\left(t-\tau \right)g\left(\tau \right)\frac{F\left(T-t\right)}{F\left(T-t+\tau \right)}$$

*R(t)* is parameterised using formula (1), *τ* is the generation time, *g(τ)* is the probability mass function (PMF) for *τ*, and *F(.)* is the cumulative mass function for the time from infection to report (for real-time modelling). To select the best-fit model, the Akaike information criterion (AIC) was computed as:


(4) $$AIC=2k-2\ln\;\left(L\right)$$

The value of AIC depends on *k*, the number of parameters in the model, and *L*, the value of the log-likelihood function calculated using formula (2). The model with the smallest AIC value was deemed the best-fit model.

#### Counterfactual interference

We simulated a counterfactual scenario, assuming that holidays did not exist, and calculated how *R_t_* would be expected to change in such a scenario. First, we estimated *R_t_* using the best-fit model. Second, we forced the holiday to be equal to 0 throughout the entire study period and reran the best-fit model to generate the counterfactual *R_t_*. We calculated the mean of estimated *R_t_* and counterfactual *R_t_* for different types of holidays among the 4 prefectures. The difference was calculated as the mean of estimated *R_t_* minus the mean of counterfactual *R_t_*. Third, we back-projected the number of confirmed cases using *R_t_* estimated in 2 scenarios and calculated the differences. Fourth, we summarised the mean differences of *R_t_* and the total difference in the number of confirmed cases. The absolute increased number in each holiday and the average percentage change in *R_t_* in each prefecture were also inferred.

### Ethics statement

Our study examined data all from publicly available data source. The COVID-19 data used in this study are available from the Ministry of Health, Labour and Welfare, Japan (https://covid19.mhlw.go.jp/en/). Prior to our analysis, these datasets were rigorously de-identified and fully anonymized. Because our study did not involve identifiable personal information, it was exempt from ethical review.

## RESULTS

Table 1 summarises the public holidays and vacations in Japan during the study period. From February 15, 2020 to September 30, 2021, there were a total of 23 holiday periods. Among these, there were eight 1-day holidays, seven 3-day consecutive holidays (C3), five 4-day consecutive holidays (C4), and three 5-day consecutive holidays (NY and GW).

**Figure 1.**
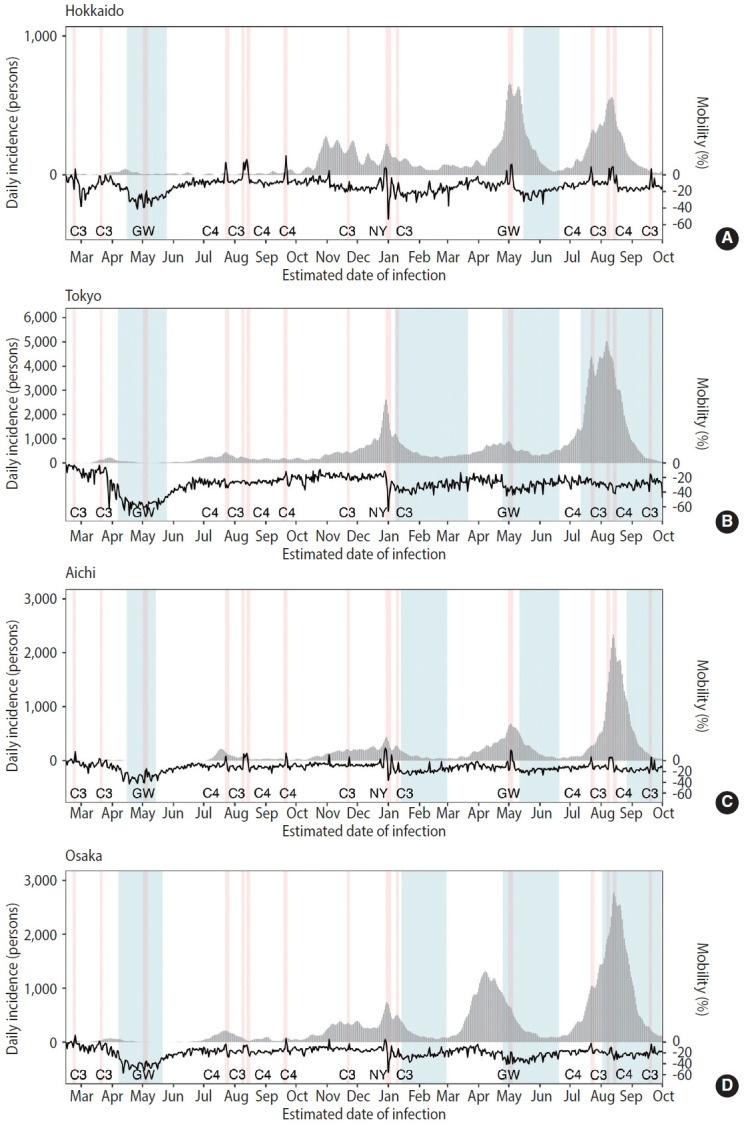
The daily incidence of confirmed coronavirus disease-19 (COVID-19) cases and the change in percentage of mobility during 2020-2021 in (A) Hokkaido, (B) Tokyo, (C) Aichi, and (D) Osaka in Japan. The data were arranged based on the estimated date of infection. Grey bars show the daily incidence of COVID-19 cases in persons, while the left y-axis represents the epidemic size. The black line indicates the percentage of change (%) in mobility compared with the baseline (the median value from the 5 weeks from January 3 to February 6, 2020) with the corresponding values displayed on the right y-axis. Red shading represents the period of public holidays. Blue shading represents the period of the statement of emergency. C3, 3-day consecutive holidays; C4, 4-day consecutive holidays; GW, Golden Week; NY, New Year, and all of these were treated as public holidays in Japan.

Figure 1 displays the temporal distribution of COVID-19 incidence and mobility in 4 prefectures. Five epidemic waves were observed in 4 prefectures from February 2020 to September 2021. In Hokkaido, the epidemic scale was relatively small as the peak incidence was 665 cases on May 5, 2021 (GW) and a state of emergency was declared twice. In Tokyo, the peak incidence was observed on August 7, 2021 (C3), with 5,024 confirmed cases, and a state of emergency was declared 4 times. In Aichi and Osaka, peak incidence took place on August 13, 2021 (C4) and August 14, 2021 (C4), with 2,348 and 2,785 confirmed cases, respectively. The mobility decreased during the study period compared with the prepandemic baseline. At most times, the mobility value was below 0, except for some holidays. Additionally, the number of confirmed cases and mobility decreased along with the state of emergency in 4 prefectures.

**Figure 2.**
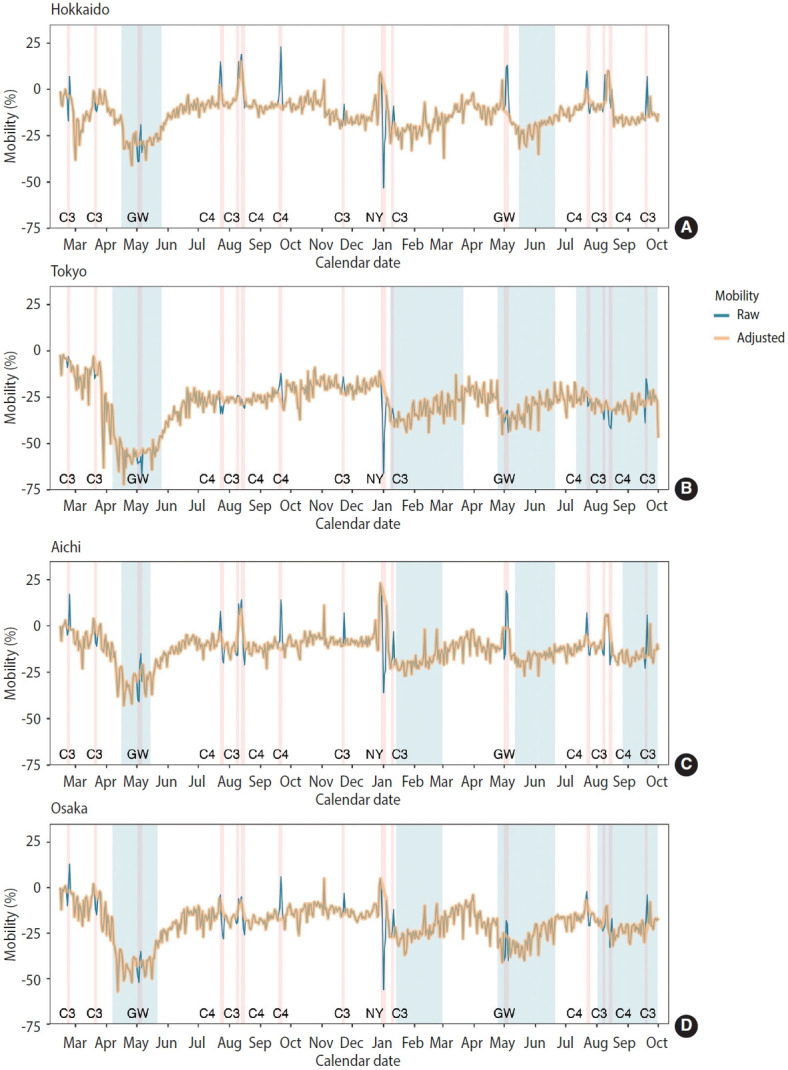
The time trend of mobility adjusted by holidays in (A) Hokkaido, (B) Tokyo, (C) Aichi, and (D) Osaka in Japan. The data were organised according to the calendar date with the y-axis representing the percent change in mobility. The blue line shows the raw mobility trend, and the orange line shows the adjusted mobility trend (trend removed during holiday periods, then imputed by linear interpolation).

To eliminate the effect of holidays on mobility, we deleted the data within the holiday periods and then imputed the gap using linear interpolation. Figure 2 compares the raw mobility and adjusted mobility over time. Although the spikes of mobility during the holiday periods were smoothed in the adjusted data compared with the raw data, the same trends were observed in both datasets.

We compared 5 models (Table 2) to describe the relationship between *R_t_* and public holidays. For all 4 prefectures, model 5, which included the state of emergency, public holidays, and adjusted mobility was selected as the best-fit model. The coefficient of public holidays was 0.06 (95% confidence interval [CI], 0.03 to 0.08) for Hokkaido, 0.03 (95% CI, 0.02 to 0.04) for Tokyo, 0.05 (95% CI, 0.03 to 0.06) for Aichi, and 0.17 (95% CI, 0.16 to 0.18) for Osaka.

In Table 3, we showed the predicted *R_t_* and confirmed cases using model 5 and calculated the counterfactual scenario. The results indicated that, if a period that was originally a holiday became a normal period, the *R_t_* would decrease by 0.06 (95% CI, 0.04 to 0.11) in Hokkaido, 0.03 (95% CI, 0.02 to 0.05) in Tokyo, 0.06 (95% CI, 0.04 to 0.10) in Aichi, and 0.20 (95% CI, 0.18 to 0.23) in Osaka. From the large coefficient for the holiday variable, the direct impact is interpreted as large and especially the largest in Osaka among 4 prefectures we examined. Because of the presence of holidays, the number of confirmed cases increased by 580 (95% CI, 213 to 954) in Hokkaido, 2,209 (95% CI, 1,230 to 3,201) in Tokyo, 1,086 (95% CI, 478 to 1,686) in Aichi, and 5,211 (95% CI, 4,554 to 5,867) in Osaka.

**Figure 3.**
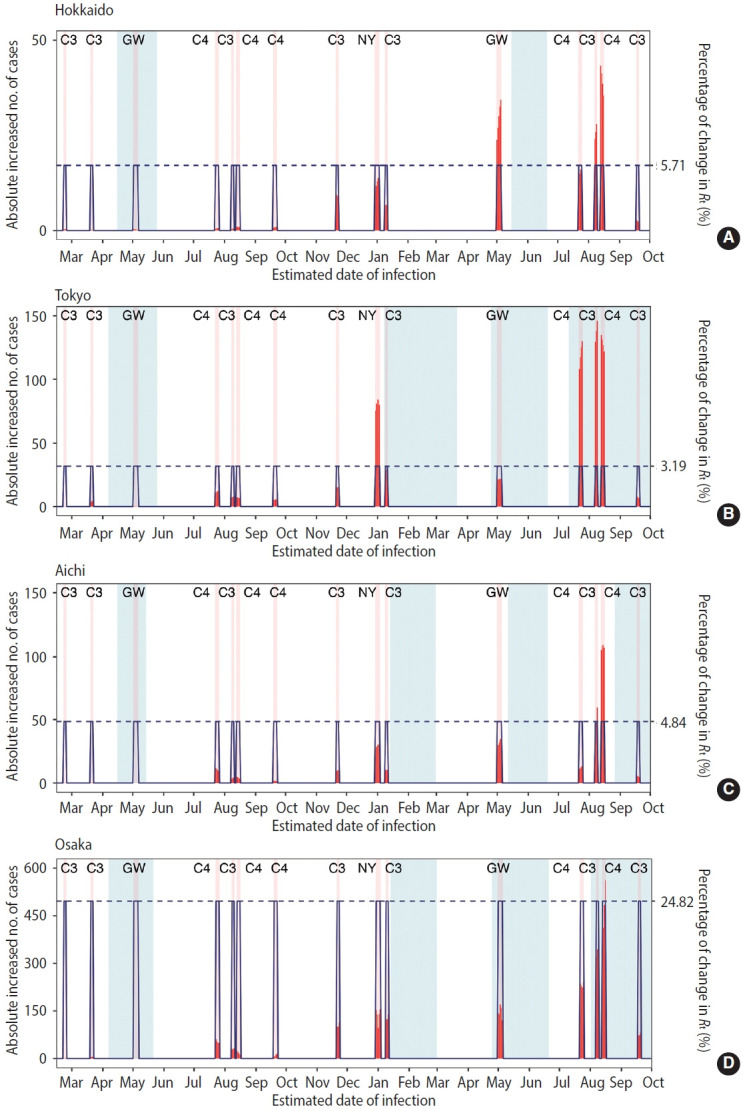
Changes in effective reproduction number (*R_t_*) and daily incidence of coronavirus disease-19 (COVID-19) between (A) Hokkaido, (B) Tokyo, (C) Aichi, and (D) Osaka in Japan. The data were organised based on the estimated date of infection. The difference between the mean estimated *R_t_* and the mean counterfactual *R_t_* for different types of holidays was calculated as “Percentage of change in *R_t_* (%).” We used solid blue lines to show the average increase of *R_t_* compared with the counterfactual scenario. The horizontal dotted line represents the corresponding value. Dark red bars represent the increase in the absolute number of confirmed COVID-19 cases in different public holidays.

The increased number of cases associated with each holiday and the percentage of change in *R_t_* in 4 prefectures are shown in Figure 3. The increase in the absolute number of cases depended on whether the public holiday appeared within the epidemic waves and the overall scale of the epidemic wave. The presence of holidays intensified transmission by 5.71% in Hokkaido, 3.19% in Tokyo, 4.84% in Aichi, and 24.82% in Osaka.

## DISCUSSION

The current study examined the relationship between consecutive public holidays and COVID-19 transmission in Japan. Modelling *R_t_* as a function of mobility and public holidays, we successfully reconstructed the observed number of confirmed cases in 4 urban prefectures. Our analysis revealed the epidemiological impact of public holidays on increasing the transmission of COVID-19. Because of the different timing of public holidays in relation to the course of epidemic and because of the different overall epidemic size, substantially different results regarding the effect of holidays were observed among the 4 prefectures. Using the log-linear regression model to predict the *R_t_* was a simple approach, but provided an effective method for quantifiably calculating the causal impact of public holidays via the computation of counterfactuals.

Overall, the results of the present study revealed that public holidays amplified the transmission of COVID-19 in Japan. Our model comparison (model 5 being the best) indicated that public holidays elevated the transmission in a direct manner, while the model adjusted for the general trend of mobility. One possible mechanism is that holidays increased the mobility rate as a direct effect, and the increased mobility could have led to increased opportunities for contact, contributing to an increased number of transmissions [24]. During the COVID-19 pandemic, the mobility rate was overall kept below the pre-COVID-19 baseline. However, people’s movements during public holidays remained high [29]. The current findings support the proposal that *R_t_* and the number of confirmed cases increase after holidays, in accord with various international studies [30-32]. The declaration of a state of emergency by the government effectively restrained people’s movement, even during holidays. A previous study by our group revealed that *R_t_* rapidly decreased to a value below 1 during a state of emergency [28]. The number of inter-prefectural travellers during holiday seasons abruptly declined during a state of emergency in Japan, possibly contributing to prevention of the spatial spread of COVID-19 [33]. Importantly, the effect of the state of emergency weakened as the declarations were repeated, as a result of fatigue [13]. Additionally, public health and social measures that reduce social contact can help attenuate transmission in holiday seasons, and early intervention greatly helps to reduce the incidence [34,35].

The effect of public holidays was estimated to differ by prefecture. The largest epidemiological impact of holidays on incidence was found in Osaka (with the coefficient for holidays estimated at 0.17), and the smallest impact was found in Tokyo (0.05 as the coefficient). The differences are attributable to different epidemic dynamics in relation to the holiday (e.g., the number of cases during holidays, types of intervention during holidays, and overall epidemic size). For instance, while the mobility data in Hokkaido (Figure 1) may look very well aligned with public holidays and more impactful than Tokyo and Osaka, the coefficient of mobility for describing *R_t_* was 0.06 for Hokkaido, with an adjustment for the general trend of mobility using model 5. During the first wave of COVID-19 in 2020, no turning point was indicated in people’s mobility around holidays from February to April 2020, both in Tokyo and Osaka [36]. Another study reported that behavioural changes during public holidays were more evident in Tokyo than in Osaka and Aichi from July to September 2021, which corresponded to the time period around the Tokyo Olympic Games [17]. Tokyo is considered to have exhibited an obvious intervention effect, in that people strictly maintained their behaviour in terms of staying at home before the holiday season in 2020, and, thus, the epidemiological impact caused by behavioural changes during the holiday period may have been weaker than that in other locations [37].

We used mobility data from Google to provide valuable insights into people’s responses to COVID-19. Several previous studies have pre-processed mobility data, such as using weekly data, smoothing the dataset using a moving average method, or decomposing the time-series data to eliminate the “noise” of holidays [14,38,39]. However, we demonstrated that fluctuation around holidays contained a signature of transient changes in people’s behaviour and was meaningful to explore in our context. The holiday-removed “adjusted” mobility dataset along with independent holiday data revealed a better fit with the epidemiological data than the raw mobility dataset. To comprehensively explain the increase in COVID-19 transmission, we believe that holidays should be included as an independent time-varying variable. We used a concise model to present the relationship between public holidays and *R_t_*, then utilised the estimated *R_t_* to back-calculate the number of confirmed cases during holidays. The model can be used to predict how many people will be infected because of holidays, which could guide public health decision-making in future preparedness planning.

Our study involved several limitations that should be acknowledged. First, we only investigated COVID-19 cases from 2020 to 2021 in Japan. The subsequent replacement of the original virus by variants and increased herd protection because of vaccination would have potentially led to different results if the study period had been longer. Second, although we analysed different types of public holidays in Japan, the weighting for each holiday was treated as identical. We ignored 1-day-only holidays, and we did not attempt to infer pre-holiday and post-holiday effects. In some cases, when 2 holiday periods are close, people’s responses may also change, even on workdays during the week between holidays. The effect of holidays on COVID-19 transmission in Japan has been demonstrated, but the current findings indicate that further studies will be required to sufficiently quantify the mechanisms of increased transmission during holidays. Third, we were unable to describe all abnormal fluctuations in the temporal patterns of *R_t_* using our simplistic model, which is likely to have missed other key explanatory variables. Although our study has successfully identified the relationship between public holidays and COVID-19 transmission, more studies need to be conducted to improve the extent of prediction.

In conclusion, public holidays in Japan played a critical role in intensifying the transmission of COVID-19. To construct future preparedness programs, implementing effective public health and social measures during holiday periods in Japan is imperative.

## Figures and Tables

**Figure f4-epih-46-e2024025:**
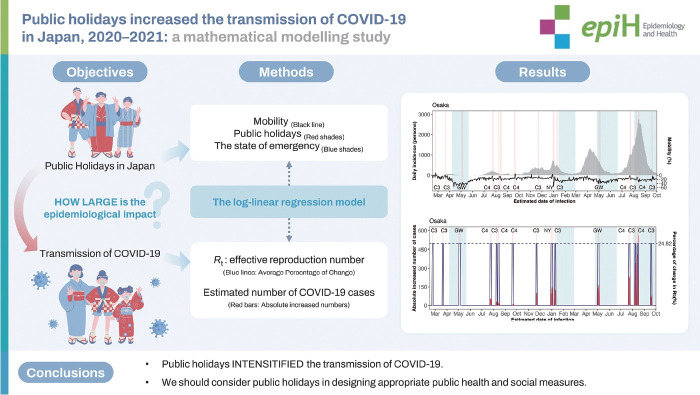


**Table 1.** Public holidays in Japan during the first to fifth waves of the pandemic, 2020-2021

Year	Time and period	Holiday’s name	Consecutive	Type
2020	Feb 11	National foundation day	No	/
	Feb 22-24	Emperor’s birthday	Yes	C3
	Mar 20-22	Vernal equinox day	Yes	C3
	Apr 29	Showa day	No	/
	May 2-6	GW (constitution memorial day+greenery day+children’s day)	Yes	GW
	Jul 23-26	Marine day+sports day	Yes	C4
	Aug 8-10	Mountain day	Yes	C3
	Aug 13-16	Obon	Yes	C4
	Sep 19-22	Respect-for-the-aged day+autumnal equinox day	Yes	C4
	Nov 3	Culture day	No	/
	Nov 21-23	Labor thanksgiving day	Yes	C3
2020-2021	Dec 30-Jan 3	NY	Yes	NY
2021	Jan 9-11	Coming-of-age day	Yes	C3
	Feb 11	National foundation day	No	/
	Feb 23	Emperor’s birthday	No	/
	Mar 20	Vernal equinox day	No	/
	Apr 29	Showa day	No	/
	May 1-5	GW (constitution memorial day+greenery day+children’s day)	Yes	GW
	Jul 22-25	Marine day+sports day	Yes	C4
	Aug 7-9	Mountain day	Yes	C3
	Aug 13-16	Obon	Yes	C4
	Sep 18-20	Respect-for-the-aged day	Yes	C3
	Sep 23	Autumnal equinox day	No	/

C3, 3-day consecutive holidays; C4, 4-day consecutive holidays; GW, Golden Week; NY, New Year, and all of these were treated as public holidays in Japan.

**Table 2.** Parameter estimates of models that predicted the effective reproduction number of COVID-19 in Hokkaido, Tokyo, Aichi, and Osaka in Japan, 2020-2021

Model	Variables	Hokkaido	AIC	Tokyo	AIC	Aichi	AIC	Osaka	AIC
Model 1	*R*	1.26 (1.24, 1.27)	9,606.90	1.71 (1.69, 1.73)	30,652.20	1.33 (1.31, 1.34)	12,767.87	1.40 (1.38, 1.41)	17,810.50
	State of emergency	-0.35 (-0.38, -0.32)		-0.04 (-0.05, -0.03)		-0.46 (-0.47, -0.44)		-0.17 (-0.18, -0.16)	
	Raw mobility	0.01 (0.01, 0.02)		0.02 (0.02, 0.02)		0.01 (0.01, 0.01)		0.01 (0.01, 0.01)	
Model 2	*R*	1.33 (1.31, 1.35)	9,462.10	1.97 (1.94, 2.00)	28,685.38	1.34 (1.33, 1.36)	12,068.29	1.47 (1.45, 1.49)	17,199.82
	State of emergency	-0.31 (-0.34, -0.28)		0.00 (-0.01, 0.01)		-0.40 (-0.42, -0.39)		-0.13 (-0.14, -0.12)	
	Adjusted mobility^1^	0.02 (0.02, 0.02)		0.03 (0.02, 0.03)		0.02 (0.02, 0.02)		0.02 (0.01, 0.02)	
Model 3	*R*	1.03 (1.02 ,1.04)	10,346.68	1.11 (1.11 ,1.12)	35,187.20	1.10 (1.09, 1.10)	13,793.42	1.12 (1.11, 1.13)	17,851.36
	State of emergency	-0.50 (-0.53, -0.47)		-0.17 (-0.18, -0.17)		-0.53 (-0.55, -0.52)		-0.28 (-0.29, -0.27)	
	Holidays	0.17 (0.14, 0.19)		0.04 (0.03, 0.05)		0.18 (0.17, 0.20)		0.21 (0.20, 0.22)	
Model 4	*R*	1.24 (1.22, 1.26)	9,601.80	1.78 (1.76, 1.80)	29,621.44	1.27 (1.26, 1.28)	12,432.13	1.37 (1.35, 1.39)	16,555.76
	State of emergency	-0.35 (-0.38, -0.32)		-0.03 (-0.04, -0.03)		-0.43 (-0.45, -0.42)		-0.18 (-0.19, -0.17)	
	Raw mobility	0.01 (0.01, 0.01)		0.02 (0.02, 0.02)		0.01 (0.01, 0.01)		0.01 (0.01, 0.01)	
	Holidays	0.03 (0.01, 0.06)		0.14 (0.14, 0.15)		0.15 (0.13, 0.16)		0.22 (0.21, 0.23)	
Model 5	*R*	1.30 (1.28, 1.32)	9,441.35	1.96 (1.93, 1.99)	28,632.59	1.32 (1.30, 1.33)	12,038.73	1.40 (1.38, 1.42)	16,437.26
	State of emergency	-0.31 (-0.34, -0.28)		0.00 (-0.01, 0.01)		-0.40 (-0.42, -0.38)		-0.15 (-0.16, -0.14)	
	Adjusted mobility^1^	0.02 (0.02, 0.02)		0.03 (0.02, 0.03)		0.02 (0.02, 0.02)		0.01 (0.01, 0.01)	
	Holidays	0.06 (0.03, 0.08)		0.03 (0.02, 0.04)		0.05 (0.03, 0.06)		0.17 (0.16, 0.18)	

Values are presented as parameter (95% confidence interval). COVID-19, coronavirus disease 2019; AIC, Akaike information criterion; R, reproduction number.

1 Adjusted mobility: data during holidays were removed, then imputed by linear interpolation.

**Table 3.** Differences in estimated effective reproduction number (Rt) and confirmed cases along with the estimated causal impact of public holidays on the basis of counterfactual incidence^1^

Holidays	Prefectures	Mean estimated *R*_t_ (95% CI)	Mean counter-factual *R*_t_ (95% CI)	Mean differences in *R*_t_ (95% CI)	Total no. of infected cases (95% CI)	Total counterfactual no. of infected cases (95% CI)	Total differences in the no. of infected cases (95% CI)
C3	Hokkaido	1.16 (1.13, 1.18)	1.09 (1.08, 1.11)	0.06 (0.04, 0.10)	2.536 (2,481, 2,591)	2,399 (2,371, 2,426)	137 (55, 221)
	Tokyo	1.20 (1.19, 1.21)	1.16 (1.15, 1.17)	0.03 (0.02, 0.05)	19,513 (19,351, 19,675)	18,909 (18,808, 19,007)	604 (344, 867)
	Aichi	1.16 (1.14, 1.18)	1.11 (1.10, 1.12)	0.05 (0.04, 0.08)	5,041 (4,964, 5,116)	4,808 (4,759, 4,858)	233 (107, 357)
	Osaka	1.32 (1.30, 1.33)	1.11 (1.10, 1.12)	0.21 (0.19, 0.23)	9,985 (9,857, 10,113)	8,424 (8,351, 8,498)	1,561 (1,359, 1,763)
C4	Hokkaido	1.32 (1.29, 1.35)	1.25 (1.23, 1.27)	0.07 (0.04, 0.12)	4,269 (4,180, 4,360)	4,038 (3,972, 4,106)	231 (75, 388)
	Tokyo	1.04 (1.03, 1.05)	1.01 (1.00, 1.01)	0.03 (0.02, 0.04)	35,352 (35,061, 35,643)	34,258 (34,071, 34,439)	1,094 (623, 1,572)
	Aichi	1.23 (1.21, 1.25)	1.17 (1.16, 1.18)	0.06 (0.04, 0.08)	11,828 (11,662, 11,991)	11,282 (11,179, 11,386)	546 (276, 812)
	Osaka	1.30 (1.28, 1.32)	1.10 (1.09, 1.10)	0.20 (0.19, 0.23)	15,044 (14,860, 15,227)	12,692 (12,601, 12,783)	2,352 (2,078, 2,626)
GW	Hokkaido	0.85 (0.83, 0.88)	0.81 (0.79, 0.82)	0.05 (0.02, 0.08)	2,762 (2,706, 2,821)	2,613 (2,588, 2,638)	149 (68, 233)
	Tokyo	0.69 (0.68, 0.69)	0.66 (0.66, 0.67)	0.02 (0.01, 0.03)	3,508 (3,478, 3,538)	3,399 (3,380, 3,418)	109 (60, 157)
	Aichi	0.97 (0.96, 0.99)	0.93 (0.92, 0.94)	0.04 (0.03, 0.07)	3,496 (3,450, 3,542)	3,335 (3,301, 3,370)	161 (80, 241)
	Osaka	0.89 (0.87, 0.90)	0.75 (0.74, 0.76)	0.14 (0.13, 0.16)	3,708 (3,664, 3,752)	3,129 (3,107, 3,150)	580 (514, 645)
NY	Hokkaido	1.45 (1.42, 1.48)	1.37 (1.34, 1.40)	0.08 (0.05, 0.14)	1,171 (1,145, 1,197)	1,107 (1,086, 1,130)	63 (15, 112)
	Tokyo	1.27 (1.26, 1.28)	1.23 (1.22, 1.24)	0.03 (0.02, 0.05)	13,020 (12,899, 13,143)	12,617 (12,538, 12,694)	403 (205, 605)
	Aichi	1.86 (1.82, 1.90)	1.78 (1.74, 1.82)	0.09 (0.05, 0.16)	3,163 (3,101, 3,226)	3,017 (2,950, 3,086)	146 (15, 276)
	Osaka	1.64 (1.61, 1.66)	1.38 (1.36, 1.40)	0.26 (0.23, 0.30)	4,592 (4,525, 4,661)	3,874 (3,827, 3,922)	718 (603, 834)
Total	Hokkaido			0.06 (0.04, 0.11)			580 (213, 954)
	Tokyo			0.03 (0.02, 0.05)	-	-	2,209 (1,230, 3,201)
	Aichi	-		0.06 (0.04, 0.10)			1,086 (478, 1,686)
	Osaka	-	-	0.20 (0.18, 0.23)	-	-	5,211 (4,554, 5,867)

CI, confidence interval; C3, 3-day consecutive holidays; C4, 4-day consecutive holidays; GW, Golden Week; NY, New Year, and all of these were treated as public holidays in Japan.

1 The mean estimated Rt was calculated by the sum of Rt in a holiday type divided by the number of days of the holiday; The counterfactual outcome was to set the value of the holiday variable as 0; The total number of infected cases was the total number of infected cases in all holidays in our study period.

## Supplementary materials

Supplementary Material 1. The holiday data used in the present study
